# Primitive bilateral nipple necrosis: a case report

**DOI:** 10.11604/pamj.2014.19.107.5221

**Published:** 2014-09-30

**Authors:** Ihssane Hakimi, Driss Moussaoui

**Affiliations:** 1 Department of Gynecology-Obstetrics, Military Hospital of Instruction Mohammed V, Rabat, Morocco

**Keywords:** Necrosis, plastic surgery, etiology

## Image in medicine

Nipple necrosis is a usual complication after breast plastic surgery, however a spontaneous necrosis of the nipple is exceptional and especially without any apparent etiology. We searched in all accessible databases without finding a similar case in the literature. It's a about a 14 years old girl, refugee in Zaatari camp in Jordan. In her medical history, we found a purulent pleurisy of atypical germs treated two years ago, she presented in the last two months a spontaneous bilateral nipple necrosis (A et B). Physical examination showed no pubertal or gynecologic abnormalities. Breast ultrasound was normal. A hormonal balance and an assessment of vacuities were without abnormalities. Otherwise, a Chest -X-Rays was normal. Histopathological examination of a nipple biopsy revealed a necrotic tissue without specific lesions. The evolution was marked by a spontaneous drop in both necrotic nipples (C).

**Figure 1:**
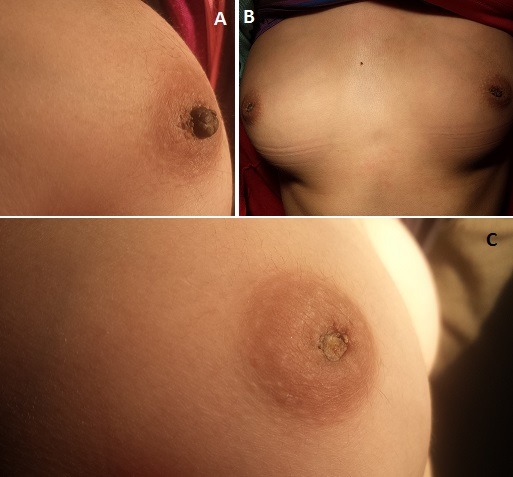
(A)picture showing a nipple necrosis; (B)bilateral nipple necrosis; (C)spontaneous drop in both necrotic nipples

